# Analysis of Stomal Bacterial Colonialization After Transcutaneous Osseointegrated Prosthetic Systems Surgery

**DOI:** 10.1001/jamanetworkopen.2022.23383

**Published:** 2022-07-22

**Authors:** Marcus Örgel, Horst-Heinrich Aschoff, Ludwig Sedlacek, Tilman Graulich, Christian Krettek, Sabine Roth, Alexander Ranker

**Affiliations:** 1 Trauma Department, Hannover Medical School, Hannover, Germany; 2 Institute for Medical Microbiology and Hospital Epidemiology, Hannover Medical School, Hannover, Germany; 3 Department of Rehabilitation Medicine, Hannover Medical School, Hannover, Germany

## Abstract

This cohort study evaluates bacterial colonization of the stoma after transcutaneous osseointegrated prosthetic systems surgery.

## Introduction

Above-knee amputations are often associated with reduced quality of life. Standard socket prosthesis can restore participation in daily life, but it has limitations owing to local skin irritation and damage.^1^ Transcutaneous osseointegrated prosthetic systems (TOPS) can be used to treat amputees instead of socket prostheses (Figure). TOPS surgery is a 2-stage procedure. After implantation of the endo-fix-stem into the bone by the press-fit method, the stoma is created after 6 weeks of osseointegration.^2^ This leads to a tight and strong connection at the bone implant interface, which is necessary for successful TOPS treatment. Rehabilitation with bone-anchored implants enables osseoperception, which improves patients’ sense of grounding.^3^

**Figure.**
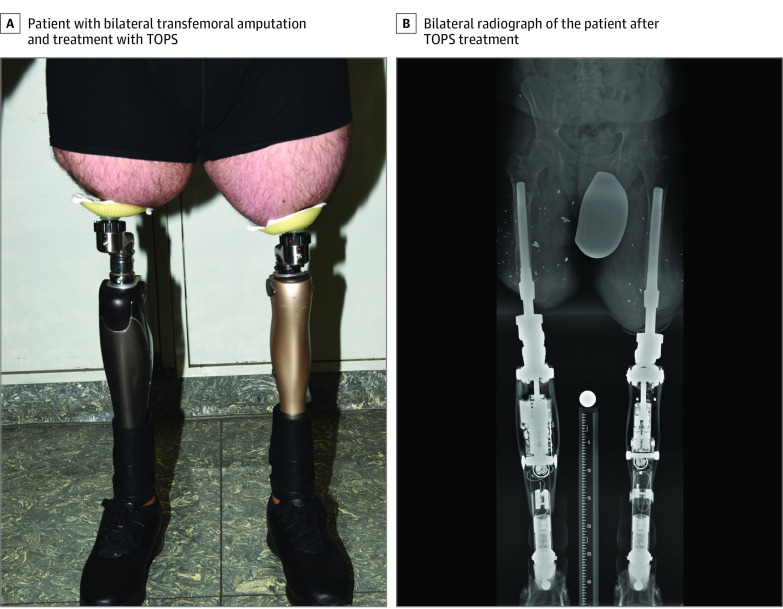
Patient With Bilateral Transfemoral Amputation and Treatment With Transcutaneous Osseointegrated Prosthetic Systems (TOPS)

Despite the advantages of TOPS, the lack of physiological skin closure is lifelong, with constant exposure of the implanted transcutaneous device to pathogens from the external environment. How normal skin flora evolves after TOPS is until now unknown. In to our knowledge, the only study on this topic, Beck et al^4^ longitudinally assessed bacteria colonizing the stoma after TOPS treatment among 10 patients. The present cohort study assessed stomal bacterial colonization after TOPS surgery.

## Methods

Between February 2017 and February 2019, we prospectively analyzed participants after TOPS treatment. The 2-step procedure was performed for every TOPS surgery by 2 surgeons.^4^ All patients received a single intravenous dose of cephazolin, 2 g, for each surgery. Local antibiotics were not used. Microbiological swab specimens obtained from the stoma were analyzed for bacterial colonization. The bacteria were classified as gram positive or gram negative. The prosthetist provided the maintenance protocol, and patients were instructed to clean their stoma with clear water daily while showering. The ethics committee of Hannover Medical School approved the study. Oral informed consent was obtained. This study followed the STROBE reporting guideline.

Death was the only exclusion criterion. Statistical analysis was performed using SPSS, version 28. Data acquisition was performed as part of our clinical protocol for follow-up of patients undergoing TOPS surgery.

## Results

TOPS surgery was performed in all 66 patients after above-knee amputation. Mean (SD) age was 50.8 (12.3) years, 29 (43.9%) were female, and mean (SD) body mass index (calculated as weight in kilograms divided by height in meters squared) was 26.9 (6.3); most amputations occurred owing to trauma. Most of the 336 bacterial isolates found on the stoma were gram positive (294 isolates [87.5%]), among which *Staphylococcus aureus*, *Staphylococcus* species, and *Streptococcus* species were most frequently encountered (Table). None of the *S aureus* isolates were oxacillin or methicillin resistant.

**Table.** Species of Bacteria Colonizing the Stoma During TOPS Surgery, the First Day After Surgery, and 3, 6, 12, and 24 Months After Surgery

Bacteria	Isolates, No. (%)						
	During surgery	Day 1	Month 3	Month 6	Month 12	Month 24	Total
All^a^	15 (4.5)	58 (17.3)	87 (25.9)	67 (19.9)	72 (21.4)	37 (11.0)	336 (100)
Gram positive^b^	14 (93.3)	56 (96.6)	76 (87.4)	55 (82.1)	62 (86.1)	31 (83.8)	294 (87.5)
Gram negative^c^	1 (6.7)	2 (3.4)	11 (12.6)	12 (17.9)	10 (13.9)	6 (16.2)	42 (12.5)

Abbreviation: TOPS, transcutaneous osseointegrated prosthetic systems.

^a^ The denominator for percentages was the total number of bacterial isolates detected.

^b^ *Staphylococcus aureus, Staphylococcus* species (*Staphylococcus lugduensis, Staphylococcus epidermidis, Staphylococcus hemolyticus,* and *Staphylococcus hominis*), *Streptococcus* species (*Streptococcus anginosus, Streptococcus agalacticae, Streptococcus dysgalacticae*, nonhemolytic and hemolytic *Streptococcus* species, and *Peptostreptococcus* species), *Corynebacterium* species, and other gram-positive species (*Bacillus cereus, Enterococcus faecalis, Cutibacterium* species, *Dermatobacter* species, *Pavimonas micra, Anaeroccocus vaginalis*, mixed flora with gram-positive bacteria, and nonspore bacteria).

^c^ *Pseudomonas* species, *Actinetobacter* species, *Enterobacter* species (*Eschericha coli, Klebsiella oxytoca,* and *Citrobacter koseri*) and other gram-negative species (*Proteus mirabilis, Serratia marescens, Morganella morganii, Fusobacterium* species, *Stenotrophomonas* species, and *Haemophilus influenza*).

## Discussion

Our results were comparable to those of Beck et al.^4^ The skin flora has several functions, such as protection against pathogen invasion, development and creation of the immune system, and catabolism of natural products.^5,6^ Among other microorganisms (eg, fungi, viruses, archaea, and arthropods), innumerable bacteria colonize the skin. Most bacteria on the stoma of patients undergoing TOPS surgery were gram positive. These findings support existing findings.^4^ In contrast with our findings, Beck et al^4^ also identified *Corynebacterium* species as one of the most common bacteria. In that study, microbiological swab specimens were obtained from the amputation site and the contralateral leg. The bacterial species found on both sides were equivalent.

In our study, we could not detect specific bacterial pathogens prospectively. We hypothesize that stable bacterial colonization occurs on the stoma. Study limitations are that short- and long-term complications and pathogens could not be derived from this work.
